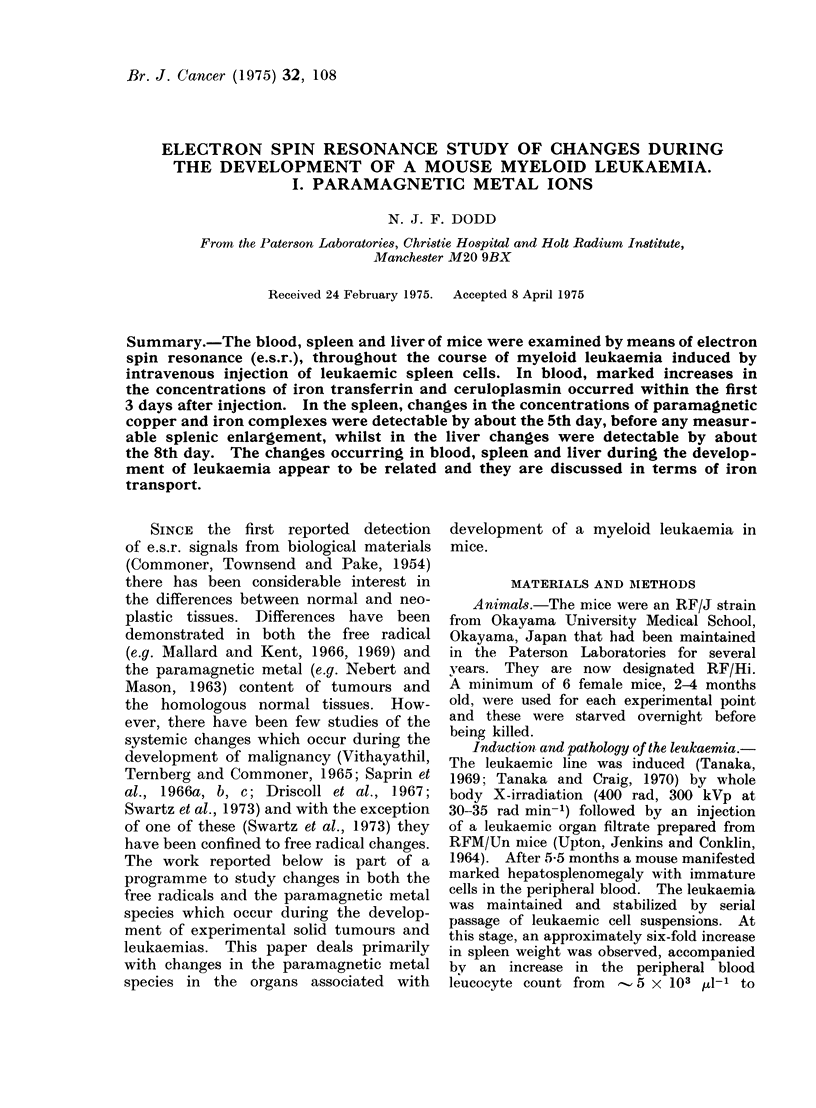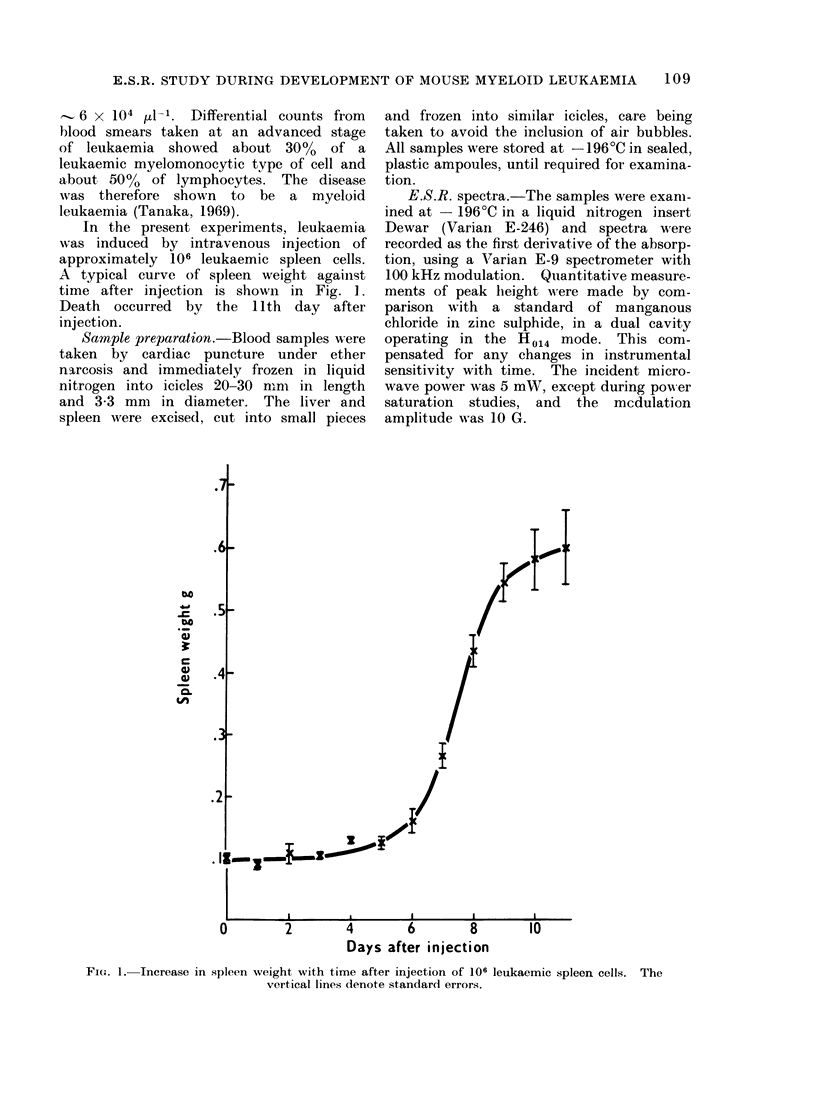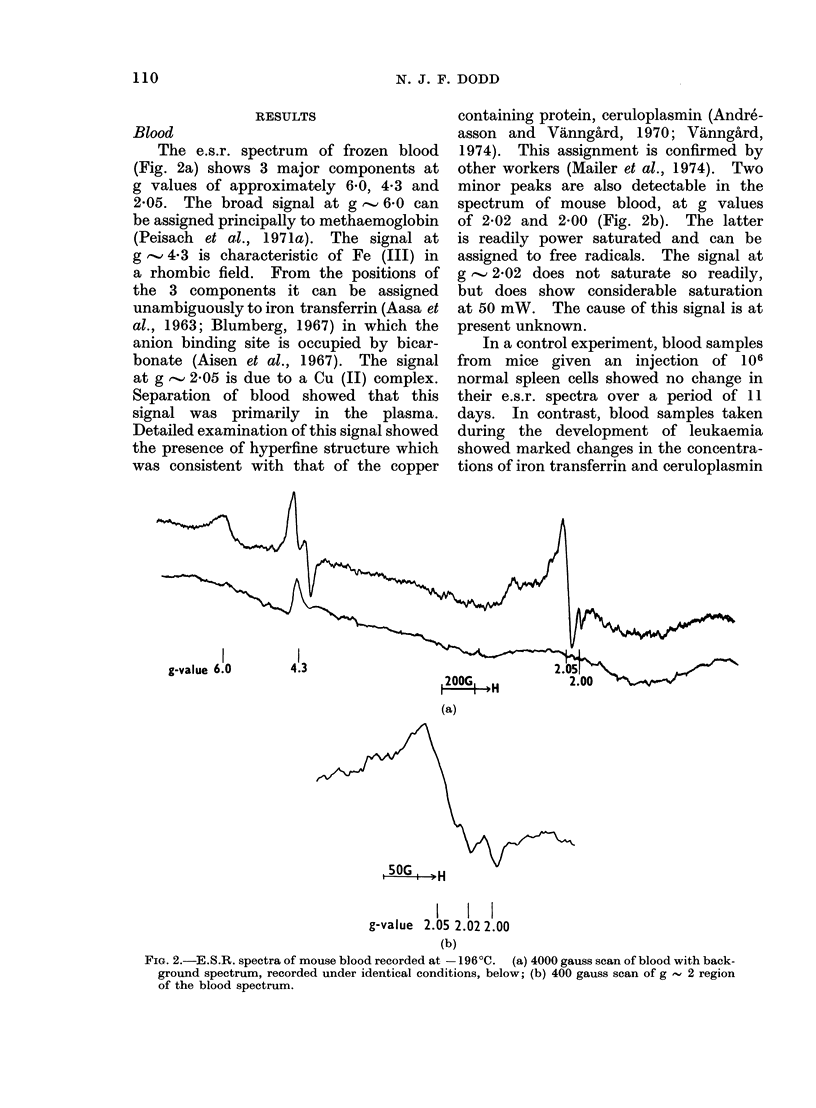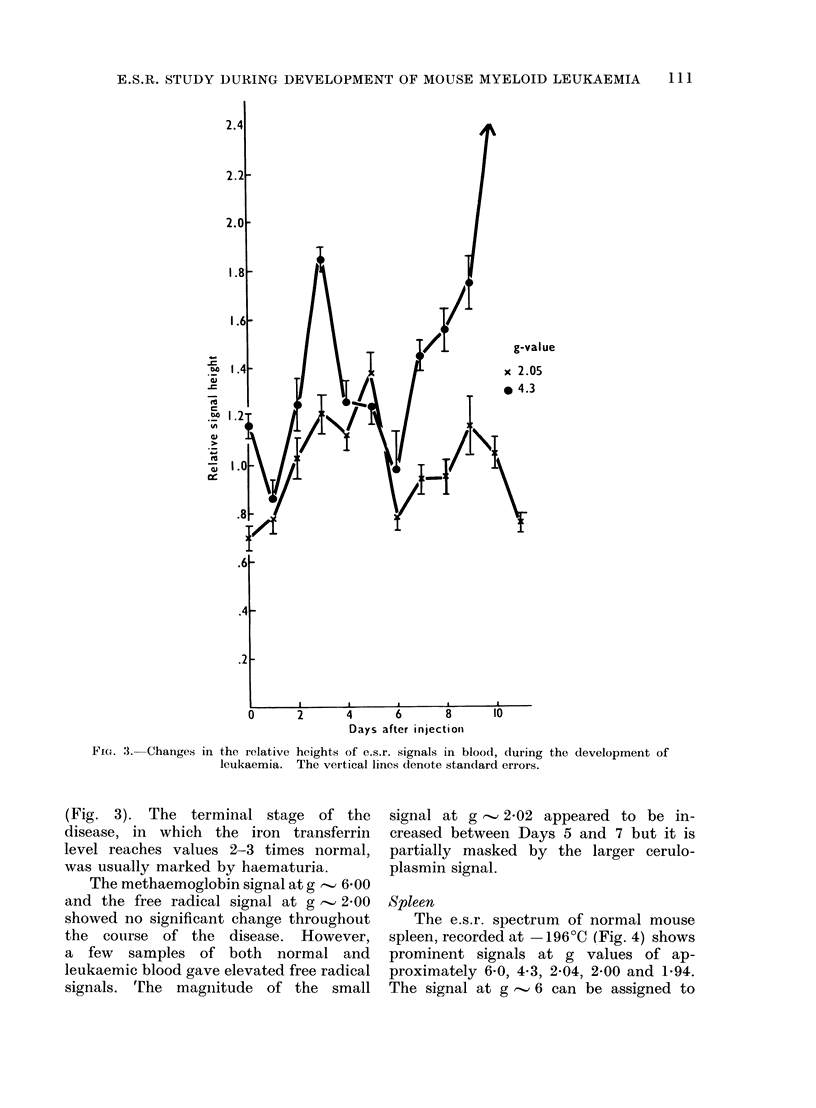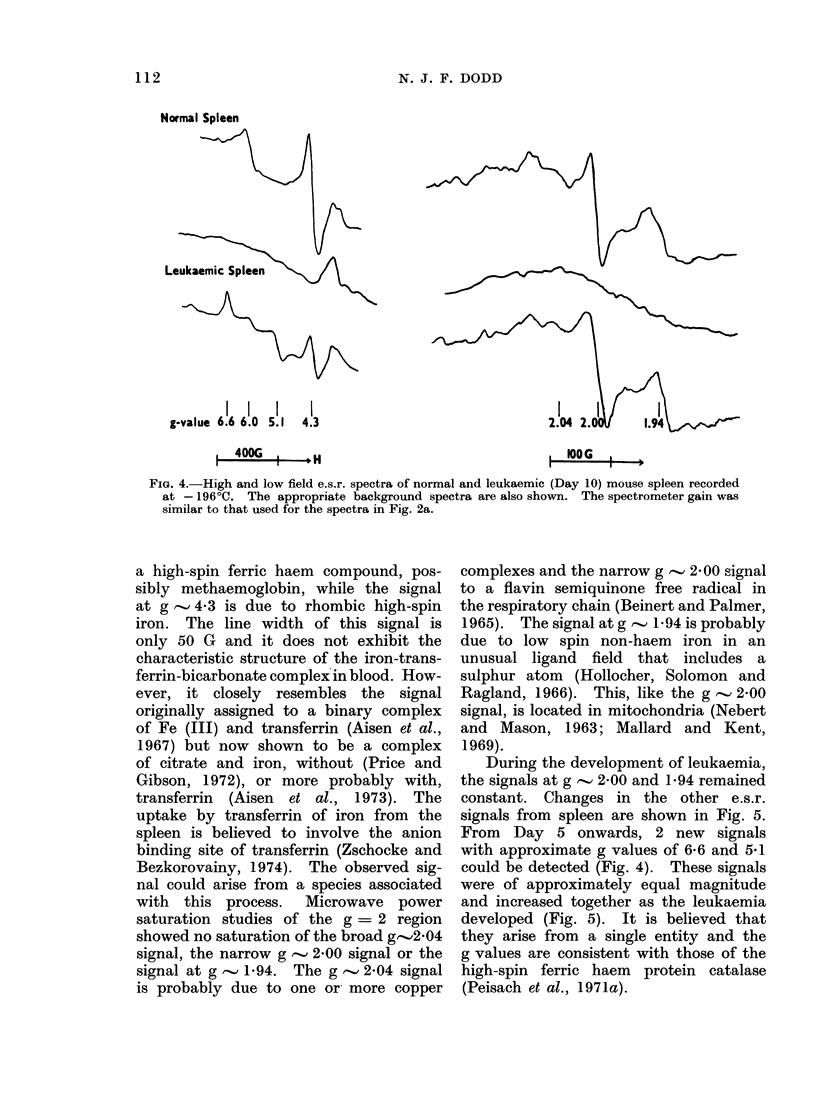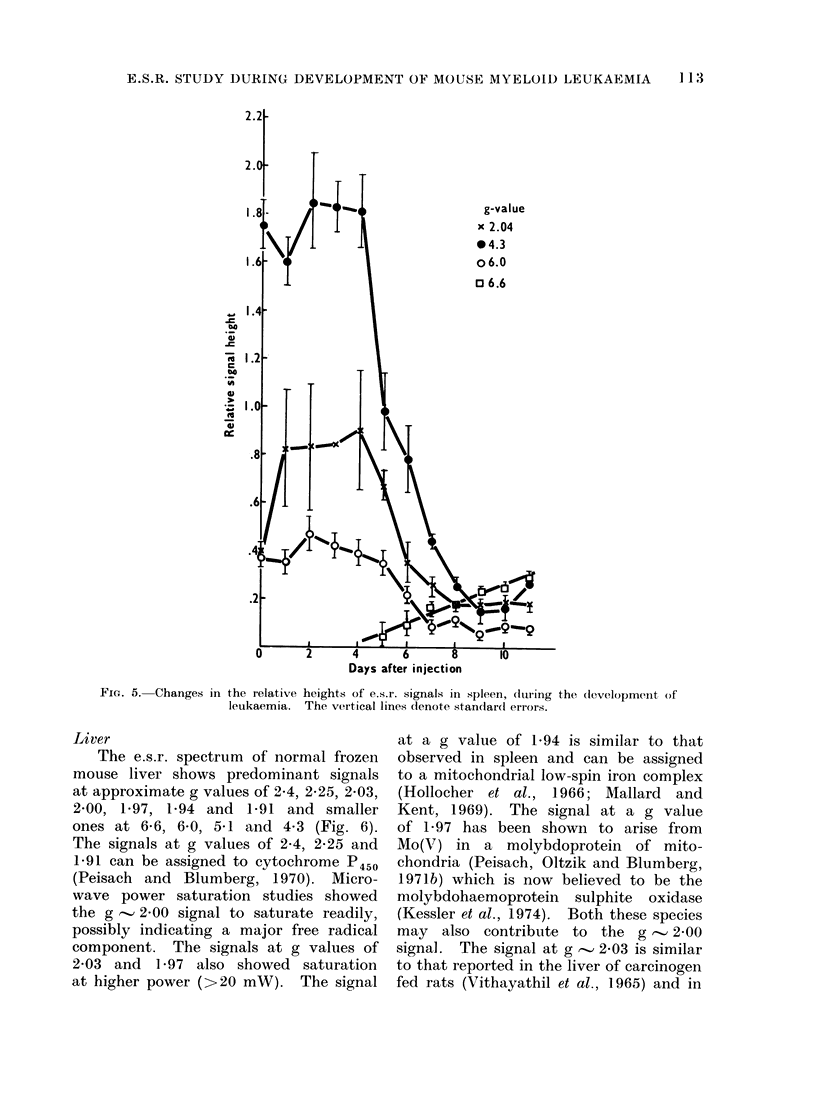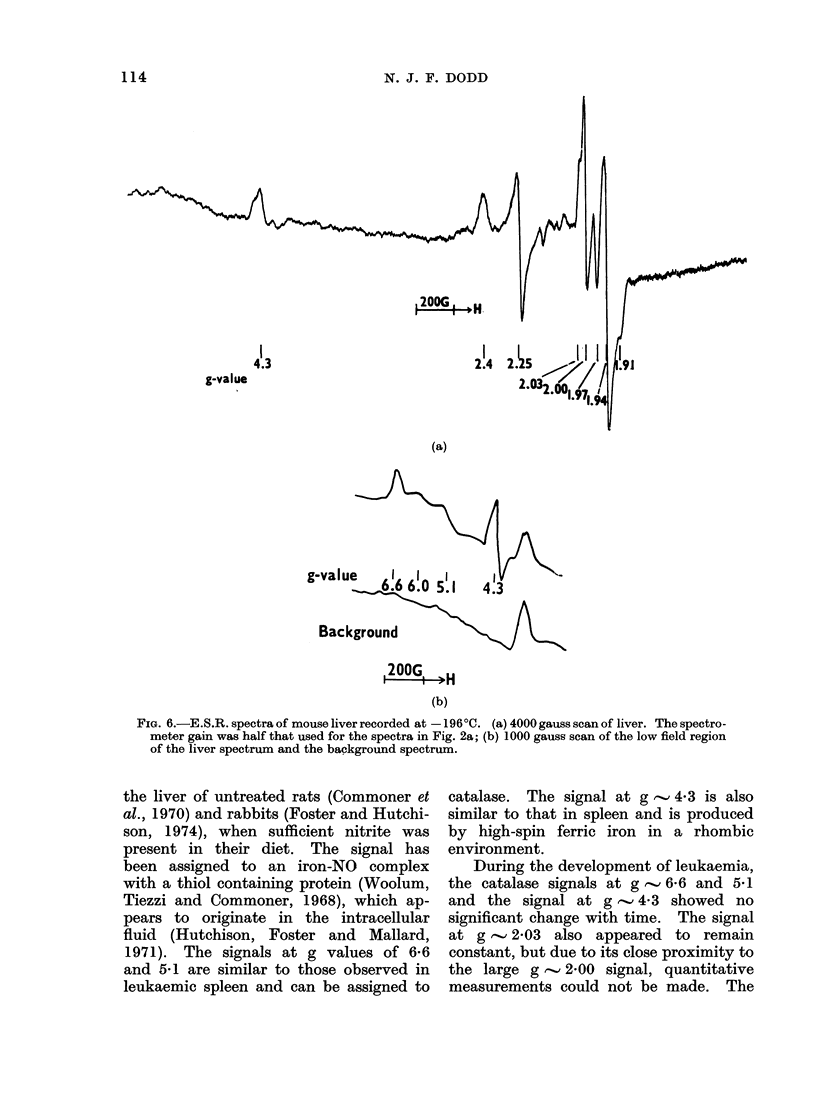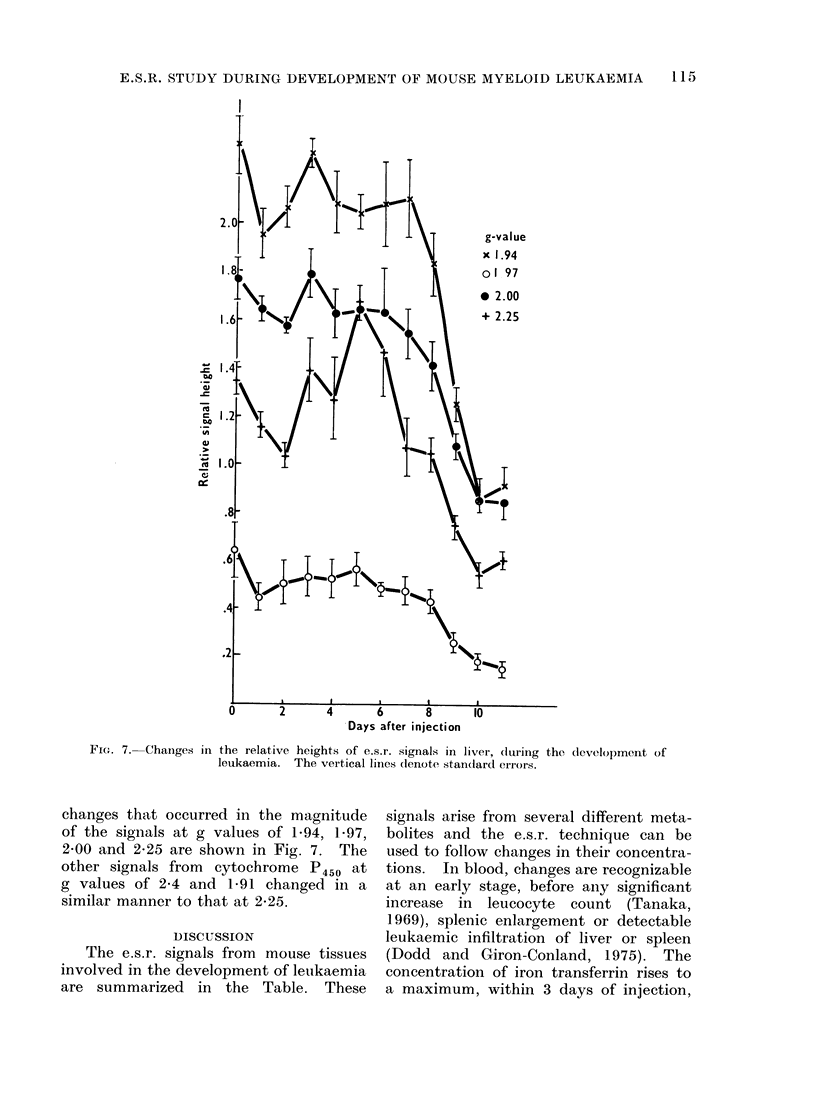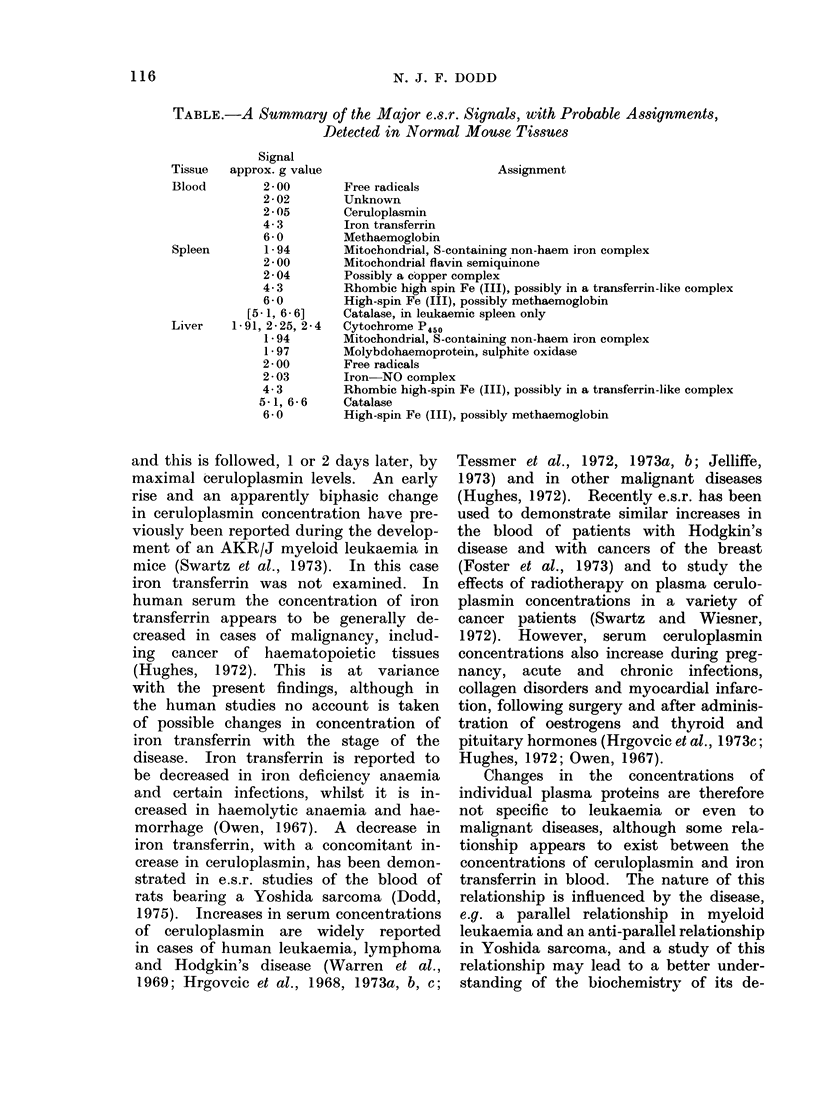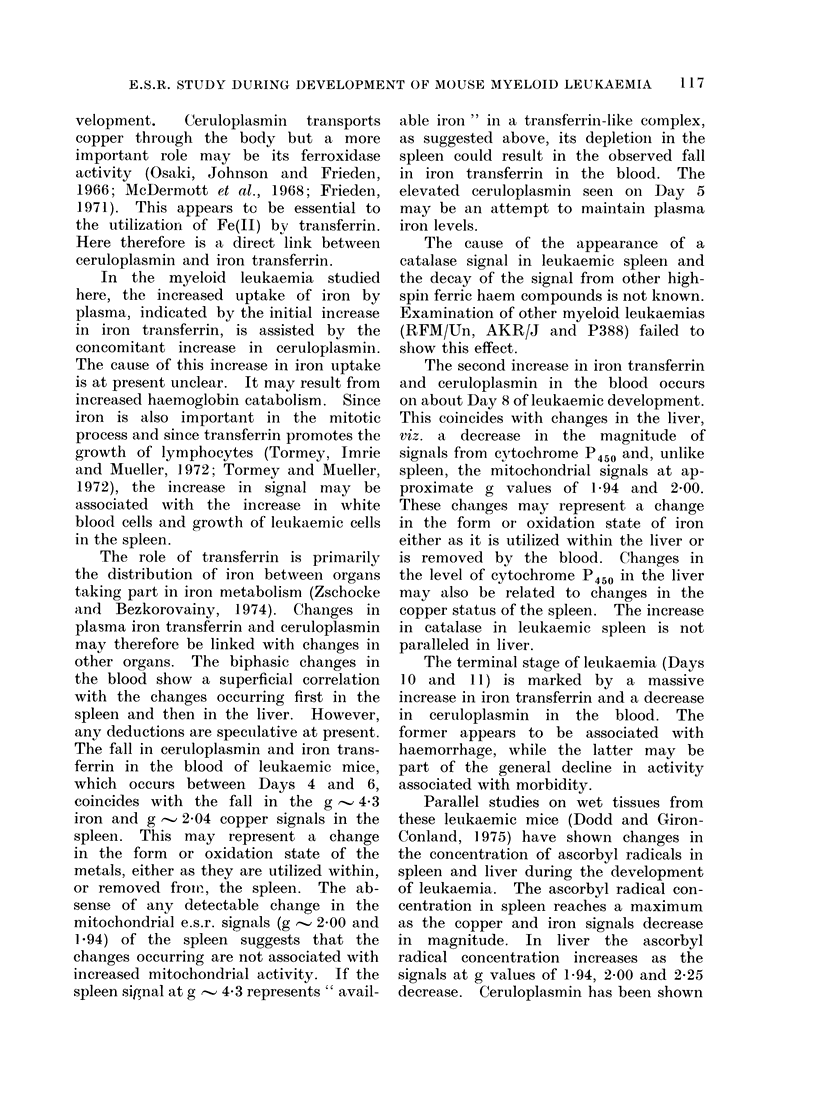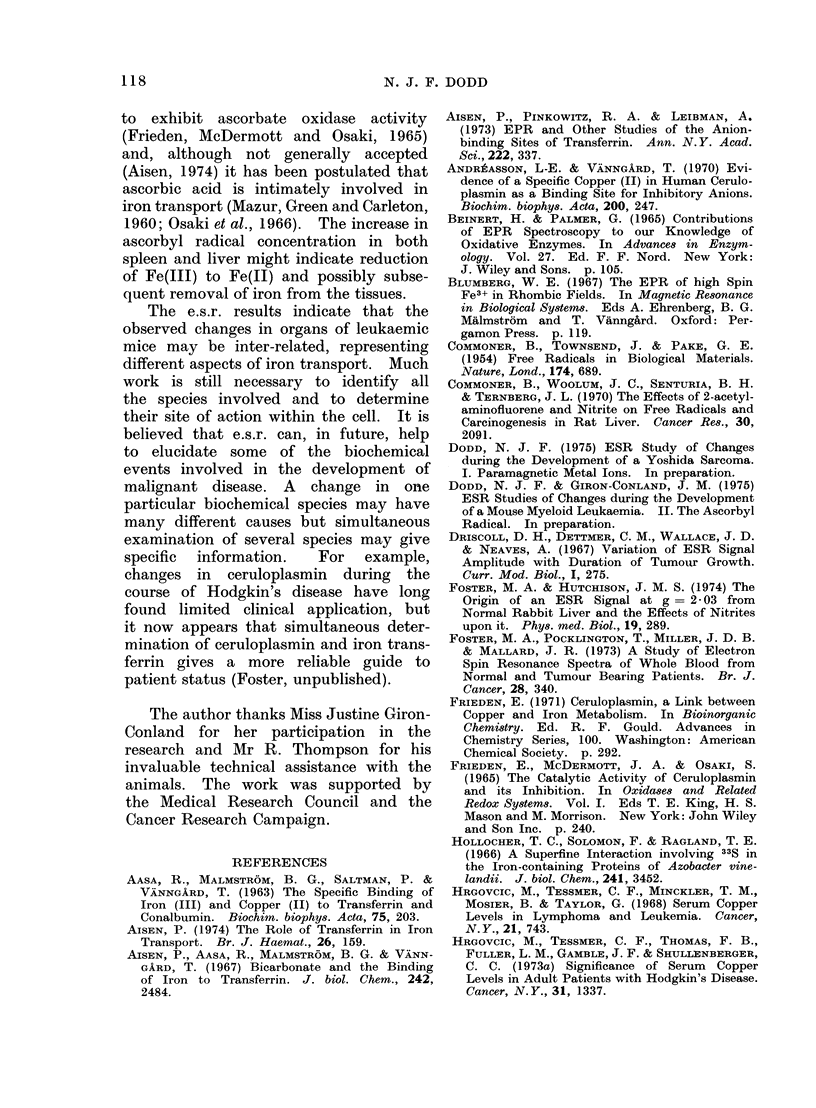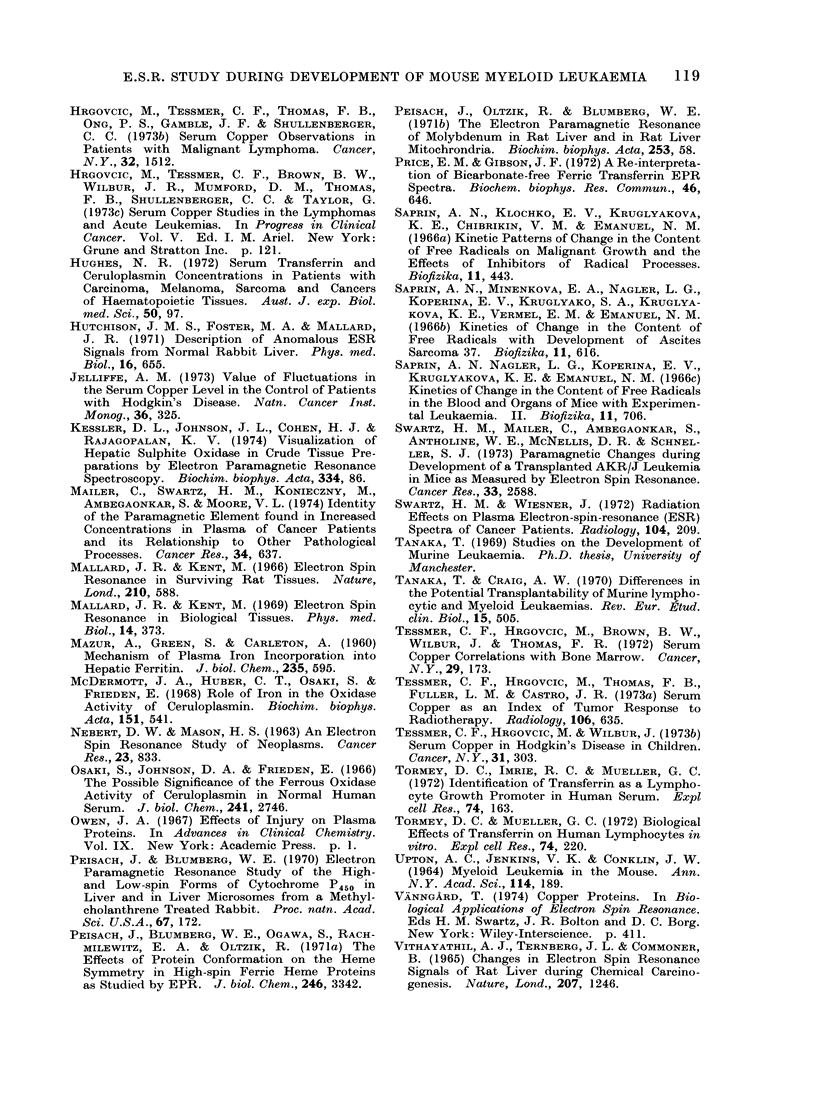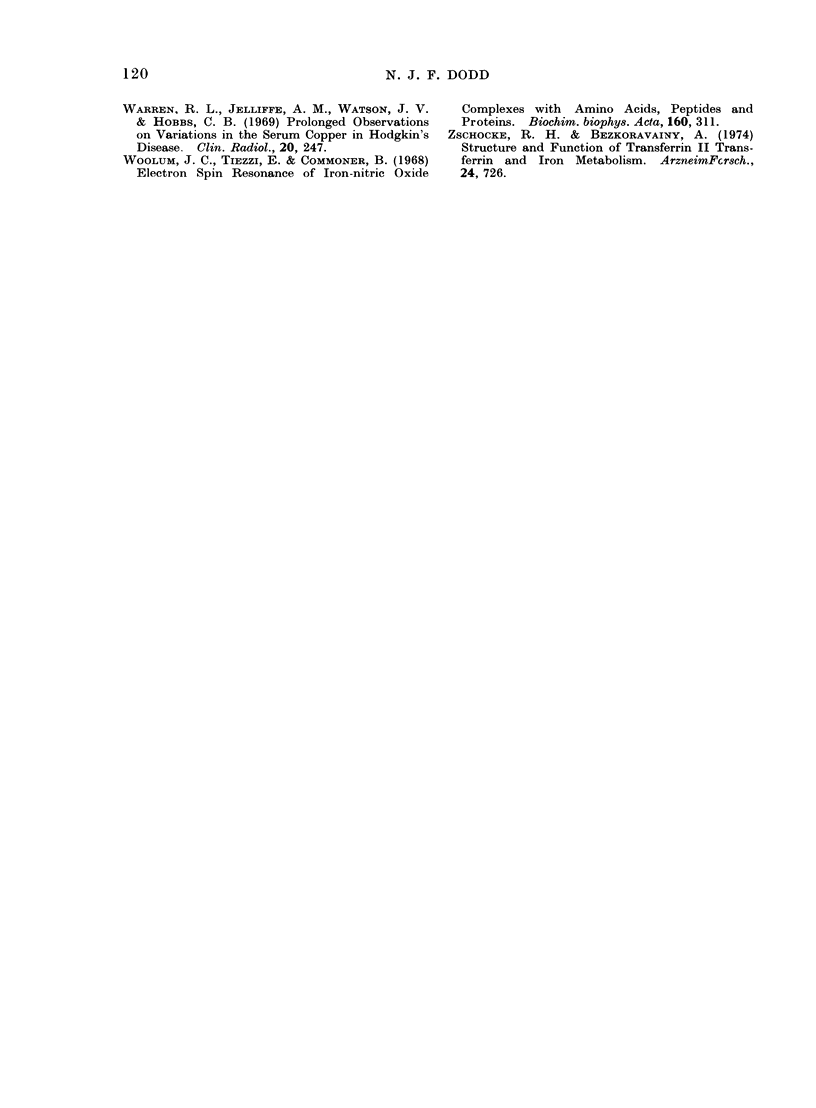# Electron spin resonance study of changes during the development of a mouse myeloid leukaemia. I. Paramagnetic metal ions.

**DOI:** 10.1038/bjc.1975.137

**Published:** 1975-07

**Authors:** N. J. Dodd

## Abstract

The blood, spleen and liver of mice were examined by means of electron spin resonance (e.s.r.), throughout the course of myeloid leukaemia induced by intravenous injection of leukaemic spleen cells. In blood, marked increases in the concentrations of iron transferrin and ceruloplasmin occurred within the first 3 days after injection. In the spleen, changes in the concentrations of paramagnetic copper and iron complexes were detectable by about the 5th day, before any measurable splenic enlargement, whilst in the liver changes were detectable by about the 8th day. The changes occurring in blood, spleen and liver during the development of leukaemia appear to be related and they are discussed in terms of iron transport.


*Br. J. Cancer* (1975) **32**, 108

### ELECTRON SPIN RESONANCE STUDY OF CHANGES DURING THE DEVELOPMENT OF A MOUSE MYELOID LEUKAEMIA. I. PARAMAGNETIC METAL IONS

N. J. F. DODD

*From the Paterson Laboratories, Christie Hospital and Holt Radium Institute, Manchester M20 9BX*

Received 24 February 1975. Accepted 8 April 1975

**Summary.—The blood, spleen and liver of mice were examined by means of electron spin resonance (e.s.r.), throughout the course of myeloid leukaemia induced by intravenous injection of leukaemic spleen cells. In blood, marked increases in the concentrations of iron transferrin and ceruloplasmin occurred within the first 3 days after injection. In the spleen, changes in the concentrations of paramagnetic copper and iron complexes were detectable by about the 5th day, before any measurable splenic enlargement, whilst in the liver changes were detectable by about the 8th day. The changes occurring in blood, spleen and liver during the development of leukaemia appear to be related and they are discussed in terms of iron transport.**

SINCE the first reported detection of e.s.r. signals from biological materials (Commoner, Townsend and Pake, 1954) there has been considerable interest in the differences between normal and neoplastic tissues. Differences have been demonstrated in both the free radical (*e.g.* Mallard and Kent, 1966, 1969) and the paramagnetic metal (*e.g.* Nebert and Mason, 1963) content of tumours and the homologous normal tissues. However, there have been few studies of the systemic changes which occur during the development of malignancy (Vithayathil, Ternberg and Commoner, 1965; Saprin *et al.*, 1966*a*, *b*, *c*; Driscoll *et al.*, 1967; Swartz *et al.*, 1973) and with the exception of one of these (Swartz *et al.*, 1973) they have been confined to free radical changes. The work reported below is part of a programme to study changes in both the free radicals and the paramagnetic metal species which occur during the development of experimental solid tumours and leukaemias. This paper deals primarily with changes in the paramagnetic metal species in the organs associated with development of a myeloid leukaemia in mice.

### MATERIALS AND METHODS

*Animals.*—The mice were an RF/J strain from Okayama University Medical School, Okayama, Japan that had been maintained in the Paterson Laboratories for several years. They are now designated RF/Hi. A minimum of 6 female mice, 2–4 months old, were used for each experimental point and these were starved overnight before being killed.

*Induction and pathology of the leukaemia.*—The leukaemic line was induced (Tanaka, 1969; Tanaka and Craig, 1970) by whole body X-irradiation (400 rad, 300 kVp at 30–35 rad $min^{-1}$) followed by an injection of a leukaemic organ filtrate prepared from RFM/Un mice (Upton, Jenkins and Conklin, 1964). After 5·5 months a mouse manifested marked hepatosplenomegaly with immature cells in the peripheral blood. The leukaemia was maintained and stabilized by serial passage of leukaemic cell suspensions. At this stage, an approximately six-fold increase in spleen weight was observed, accompanied by an increase in the peripheral blood leucocyte count from $\sim 5 \times 10^3$ $\mu l^{-1}$ to

$\sim 6 \times 10^4$ $\mu l^{-1}$. Differential counts from blood smears taken at an advanced stage of leukaemia showed about 30% of a leukaemic myelomonocytic type of cell and about 50% of lymphocytes. The disease was therefore shown to be a myeloid leukaemia (Tanaka, 1969).

In the present experiments, leukaemia was induced by intravenous injection of approximately $10^6$ leukaemic spleen cells. A typical curve of spleen weight against time after injection is shown in Fig. 1. Death occurred by the 11th day after injection.

*Sample preparation.*—Blood samples were taken by cardiac puncture under ether narcosis and immediately frozen in liquid nitrogen into icicles 20–30 mm in length and 3·3 mm in diameter. The liver and spleen were excised, cut into small pieces and frozen into similar icicles, care being taken to avoid the inclusion of air bubbles. All samples were stored at −196°C in sealed, plastic ampoules, until required for examination.

*E.S.R.* spectra.—The samples were examined at − 196°C in a liquid nitrogen insert Dewar (Varian E-246) and spectra were recorded as the first derivative of the absorption, using a Varian E-9 spectrometer with 100 kHz modulation. Quantitative measurements of peak height were made by comparison with a standard of manganous chloride in zinc sulphide, in a dual cavity operating in the $H_{014}$ mode. This compensated for any changes in instrumental sensitivity with time. The incident microwave power was 5 mW, except during power saturation studies, and the modulation amplitude was 10 G.

FIG. 1.—Increase in spleen weight with time after injection of $10^6$ leukaemic spleen cells. The vertical lines denote standard errors.

RESULTS

*Blood*

The e.s.r. spectrum of frozen blood (Fig. 2a) shows 3 major components at g values of approximately 6·0, 4·3 and 2·05. The broad signal at g $\sim$ 6·0 can be assigned principally to methaemoglobin (Peisach *et al.*, 1971*a*). The signal at g $\sim$ 4·3 is characteristic of Fe (III) in a rhombic field. From the positions of the 3 components it can be assigned unambiguously to iron transferrin (Aasa *et al.*, 1963; Blumberg, 1967) in which the anion binding site is occupied by bicarbonate (Aisen *et al.*, 1967). The signal at g $\sim$ 2·05 is due to a Cu (II) complex. Separation of blood showed that this signal was primarily in the plasma. Detailed examination of this signal showed the presence of hyperfine structure which was consistent with that of the copper containing protein, ceruloplasmin (Andréasson and Vänngård, 1970; Vänngård, 1974). This assignment is confirmed by other workers (Mailer *et al.*, 1974). Two minor peaks are also detectable in the spectrum of mouse blood, at g values of 2·02 and 2·00 (Fig. 2b). The latter is readily power saturated and can be assigned to free radicals. The signal at g $\sim$ 2·02 does not saturate so readily, but does show considerable saturation at 50 mW. The cause of this signal is at present unknown.

In a control experiment, blood samples from mice given an injection of $10^6$ normal spleen cells showed no change in their e.s.r. spectra over a period of 11 days. In contrast, blood samples taken during the development of leukaemia showed marked changes in the concentrations of iron transferrin and ceruloplasmin

FIG. 2.—E.S.R. spectra of mouse blood recorded at −196°C. (a) 4000 gauss scan of blood with background spectrum, recorded under identical conditions, below; (b) 400 gauss scan of g $\sim$ 2 region of the blood spectrum.

FIG. 3.—Changes in the relative heights of e.s.r. signals in blood, during the development of leukaemia. The vertical lines denote standard errors.

(Fig. 3). The terminal stage of the disease, in which the iron transferrin level reaches values 2–3 times normal, was usually marked by haematuria.

The methaemoglobin signal at g ~ 6·00 and the free radical signal at g ~ 2·00 showed no significant change throughout the course of the disease. However, a few samples of both normal and leukaemic blood gave elevated free radical signals. The magnitude of the small signal at g ~ 2·02 appeared to be increased between Days 5 and 7 but it is partially masked by the larger ceruloplasmin signal.

*Spleen*

The e.s.r. spectrum of normal mouse spleen, recorded at −196°C (Fig. 4) shows prominent signals at g values of approximately 6·0, 4·3, 2·04, 2·00 and 1·94. The signal at g ~ 6 can be assigned to

FIG. 4.—High and low field e.s.r. spectra of normal and leukaemic (Day 10) mouse spleen recorded at $-196°C$. The appropriate background spectra are also shown. The spectrometer gain was similar to that used for the spectra in Fig. 2a.

a high-spin ferric haem compound, possibly methaemoglobin, while the signal at g $\sim$ 4·3 is due to rhombic high-spin iron. The line width of this signal is only 50 G and it does not exhibit the characteristic structure of the iron-transferrin-bicarbonate complex in blood. However, it closely resembles the signal originally assigned to a binary complex of Fe (III) and transferrin (Aisen *et al.*, 1967) but now shown to be a complex of citrate and iron, without (Price and Gibson, 1972), or more probably with, transferrin (Aisen *et al.*, 1973). The uptake by transferrin of iron from the spleen is believed to involve the anion binding site of transferrin (Zschocke and Bezkorovainy, 1974). The observed signal could arise from a species associated with this process. Microwave power saturation studies of the $g = 2$ region showed no saturation of the broad g$\sim$2·04 signal, the narrow g $\sim$ 2·00 signal or the signal at g $\sim$ 1·94. The g $\sim$ 2·04 signal is probably due to one or more copper complexes and the narrow g $\sim$ 2·00 signal to a flavin semiquinone free radical in the respiratory chain (Beinert and Palmer, 1965). The signal at g $\sim$ 1·94 is probably due to low spin non-haem iron in an unusual ligand field that includes a sulphur atom (Hollocher, Solomon and Ragland, 1966). This, like the g $\sim$ 2·00 signal, is located in mitochondria (Nebert and Mason, 1963; Mallard and Kent, 1969).

During the development of leukaemia, the signals at g $\sim$ 2·00 and 1·94 remained constant. Changes in the other e.s.r. signals from spleen are shown in Fig. 5. From Day 5 onwards, 2 new signals with approximate g values of 6·6 and 5·1 could be detected (Fig. 4). These signals were of approximately equal magnitude and increased together as the leukaemia developed (Fig. 5). It is believed that they arise from a single entity and the g values are consistent with those of the high-spin ferric haem protein catalase (Peisach *et al.*, 1971*a*).

FIG. 5.—Changes in the relative heights of e.s.r. signals in spleen, during the development of leukaemia. The vertical lines denote standard errors.

*Liver*

The e.s.r. spectrum of normal frozen mouse liver shows predominant signals at approximate g values of 2·4, 2·25, 2·03, 2·00, 1·97, 1·94 and 1·91 and smaller ones at 6·6, 6·0, 5·1 and 4·3 (Fig. 6). The signals at g values of 2·4, 2·25 and 1·91 can be assigned to cytochrome $P_{450}$ (Peisach and Blumberg, 1970). Microwave power saturation studies showed the g $\sim$ 2·00 signal to saturate readily, possibly indicating a major free radical component. The signals at g values of 2·03 and 1·97 also showed saturation at higher power ($>$20 mW). The signal at a g value of 1·94 is similar to that observed in spleen and can be assigned to a mitochondrial low-spin iron complex (Hollocher *et al.*, 1966; Mallard and Kent, 1969). The signal at a g value of 1·97 has been shown to arise from Mo(V) in a molybdoprotein of mitochondria (Peisach, Oltzik and Blumberg, 1971*b*) which is now believed to be the molybdohaemoprotein sulphite oxidase (Kessler *et al.*, 1974). Both these species may also contribute to the g $\sim$ 2·00 signal. The signal at g $\sim$ 2·03 is similar to that reported in the liver of carcinogen fed rats (Vithayathil *et al.*, 1965) and in

FIG. 6.—E.S.R. spectra of mouse liver recorded at −196°C. (a) 4000 gauss scan of liver. The spectrometer gain was half that used for the spectra in Fig. 2a; (b) 1000 gauss scan of the low field region of the liver spectrum and the background spectrum.

the liver of untreated rats (Commoner *et al.*, 1970) and rabbits (Foster and Hutchison, 1974), when sufficient nitrite was present in their diet. The signal has been assigned to an iron-NO complex with a thiol containing protein (Woolum, Tiezzi and Commoner, 1968), which appears to originate in the intracellular fluid (Hutchison, Foster and Mallard, 1971). The signals at g values of 6·6 and 5·1 are similar to those observed in leukaemic spleen and can be assigned to catalase. The signal at g $\sim$ 4·3 is also similar to that in spleen and is produced by high-spin ferric iron in a rhombic environment.

During the development of leukaemia, the catalase signals at g $\sim$ 6·6 and 5·1 and the signal at g $\sim$ 4·3 showed no significant change with time. The signal at g $\sim$ 2·03 also appeared to remain constant, but due to its close proximity to the large g $\sim$ 2·00 signal, quantitative measurements could not be made. The

FIG. 7.—Changes in the relative heights of e.s.r. signals in liver, during the development of leukaemia. The vertical lines denote standard errors.

changes that occurred in the magnitude of the signals at g values of 1·94, 1·97, 2·00 and 2·25 are shown in Fig. 7. The other signals from cytochrome $P_{450}$ at g values of 2·4 and 1·91 changed in a similar manner to that at 2·25.

### DISCUSSION

The e.s.r. signals from mouse tissues involved in the development of leukaemia are summarized in the Table. These signals arise from several different metabolites and the e.s.r. technique can be used to follow changes in their concentrations. In blood, changes are recognizable at an early stage, before any significant increase in leucocyte count (Tanaka, 1969), splenic enlargement or detectable leukaemic infiltration of liver or spleen (Dodd and Giron-Conland, 1975). The concentration of iron transferrin rises to a maximum, within 3 days of injection,

TABLE.—*A Summary of the Major e.s.r. Signals, with Probable Assignments, Detected in Normal Mouse Tissues*

| Tissue | Signal approx. g value | Assignment |
|---|---|---|
| Blood | 2·00 | Free radicals |
| | 2·02 | Unknown |
| | 2·05 | Ceruloplasmin |
| | 4·3 | Iron transferrin |
| | 6·0 | Methaemoglobin |
| Spleen | 1·94 | Mitochondrial, S-containing non-haem iron complex |
| | 2·00 | Mitochondrial flavin semiquinone |
| | 2·04 | Possibly a copper complex |
| | 4·3 | Rhombic high spin Fe (III), possibly in a transferrin-like complex |
| | 6·0 | High-spin Fe (III), possibly methaemoglobin |
| | [5·1, 6·6] | Catalase, in leukaemic spleen only |
| Liver | 1·91, 2·25, 2·4 | Cytochrome $P_{450}$ |
| | 1·94 | Mitochondrial, S-containing non-haem iron complex |
| | 1·97 | Molybdohaemoprotein, sulphite oxidase |
| | 2·00 | Free radicals |
| | 2·03 | Iron—NO complex |
| | 4·3 | Rhombic high-spin Fe (III), possibly in a transferrin-like complex |
| | 5·1, 6·6 | Catalase |
| | 6·0 | High-spin Fe (III), possibly methaemoglobin |

and this is followed, 1 or 2 days later, by maximal ceruloplasmin levels. An early rise and an apparently biphasic change in ceruloplasmin concentration have previously been reported during the development of an AKR/J myeloid leukaemia in mice (Swartz *et al.*, 1973). In this case iron transferrin was not examined. In human serum the concentration of iron transferrin appears to be generally decreased in cases of malignancy, including cancer of haematopoietic tissues (Hughes, 1972). This is at variance with the present findings, although in the human studies no account is taken of possible changes in concentration of iron transferrin with the stage of the disease. Iron transferrin is reported to be decreased in iron deficiency anaemia and certain infections, whilst it is increased in haemolytic anaemia and haemorrhage (Owen, 1967). A decrease in iron transferrin, with a concomitant increase in ceruloplasmin, has been demonstrated in e.s.r. studies of the blood of rats bearing a Yoshida sarcoma (Dodd, 1975). Increases in serum concentrations of ceruloplasmin are widely reported in cases of human leukaemia, lymphoma and Hodgkin's disease (Warren *et al.*, 1969; Hrgovcic *et al.*, 1968, 1973*a*, *b*, *c*; Tessmer *et al.*, 1972, 1973*a*, *b*; Jelliffe, 1973) and in other malignant diseases (Hughes, 1972). Recently e.s.r. has been used to demonstrate similar increases in the blood of patients with Hodgkin's disease and with cancers of the breast (Foster *et al.*, 1973) and to study the effects of radiotherapy on plasma ceruloplasmin concentrations in a variety of cancer patients (Swartz and Wiesner, 1972). However, serum ceruloplasmin concentrations also increase during pregnancy, acute and chronic infections, collagen disorders and myocardial infarction, following surgery and after administration of oestrogens and thyroid and pituitary hormones (Hrgovcic *et al.*, 1973*c*; Hughes, 1972; Owen, 1967).

Changes in the concentrations of individual plasma proteins are therefore not specific to leukaemia or even to malignant diseases, although some relationship appears to exist between the concentrations of ceruloplasmin and iron transferrin in blood. The nature of this relationship is influenced by the disease, *e.g.* a parallel relationship in myeloid leukaemia and an anti-parallel relationship in Yoshida sarcoma, and a study of this relationship may lead to a better understanding of the biochemistry of its development. Ceruloplasmin transports copper through the body but a more important role may be its ferroxidase activity (Osaki, Johnson and Frieden, 1966; McDermott *et al.*, 1968; Frieden, 1971). This appears to be essential to the utilization of Fe(II) by transferrin. Here therefore is a direct link between ceruloplasmin and iron transferrin.

In the myeloid leukaemia studied here, the increased uptake of iron by plasma, indicated by the initial increase in iron transferrin, is assisted by the concomitant increase in ceruloplasmin. The cause of this increase in iron uptake is at present unclear. It may result from increased haemoglobin catabolism. Since iron is also important in the mitotic process and since transferrin promotes the growth of lymphocytes (Tormey, Imrie and Mueller, 1972; Tormey and Mueller, 1972), the increase in signal may be associated with the increase in white blood cells and growth of leukaemic cells in the spleen.

The role of transferrin is primarily the distribution of iron between organs taking part in iron metabolism (Zschocke and Bezkorovainy, 1974). Changes in plasma iron transferrin and ceruloplasmin may therefore be linked with changes in other organs. The biphasic changes in the blood show a superficial correlation with the changes occurring first in the spleen and then in the liver. However, any deductions are speculative at present. The fall in ceruloplasmin and iron transferrin in the blood of leukaemic mice, which occurs between Days 4 and 6, coincides with the fall in the $g \sim 4{\cdot}3$ iron and $g \sim 2{\cdot}04$ copper signals in the spleen. This may represent a change in the form or oxidation state of the metals, either as they are utilized within, or removed from, the spleen. The absense of any detectable change in the mitochondrial e.s.r. signals ($g \sim 2{\cdot}00$ and 1·94) of the spleen suggests that the changes occurring are not associated with increased mitochondrial activity. If the spleen signal at $g \sim 4{\cdot}3$ represents " available iron " in a transferrin-like complex, as suggested above, its depletion in the spleen could result in the observed fall in iron transferrin in the blood. The elevated ceruloplasmin seen on Day 5 may be an attempt to maintain plasma iron levels.

The cause of the appearance of a catalase signal in leukaemic spleen and the decay of the signal from other high-spin ferric haem compounds is not known. Examination of other myeloid leukaemias (RFM/Un, AKR/J and P388) failed to show this effect.

The second increase in iron transferrin and ceruloplasmin in the blood occurs on about Day 8 of leukaemic development. This coincides with changes in the liver, *viz.* a decrease in the magnitude of signals from cytochrome $P_{450}$ and, unlike spleen, the mitochondrial signals at approximate g values of 1·94 and 2·00. These changes may represent a change in the form or oxidation state of iron either as it is utilized within the liver or is removed by the blood. Changes in the level of cytochrome $P_{450}$ in the liver may also be related to changes in the copper status of the spleen. The increase in catalase in leukaemic spleen is not paralleled in liver.

The terminal stage of leukaemia (Days 10 and 11) is marked by a massive increase in iron transferrin and a decrease in ceruloplasmin in the blood. The former appears to be associated with haemorrhage, while the latter may be part of the general decline in activity associated with morbidity.

Parallel studies on wet tissues from these leukaemic mice (Dodd and Giron-Conland, 1975) have shown changes in the concentration of ascorbyl radicals in spleen and liver during the development of leukaemia. The ascorbyl radical concentration in spleen reaches a maximum as the copper and iron signals decrease in magnitude. In liver the ascorbyl radical concentration increases as the signals at g values of 1·94, 2·00 and 2·25 decrease. Ceruloplasmin has been shown to exhibit ascorbate oxidase activity (Frieden, McDermott and Osaki, 1965) and, although not generally accepted (Aisen, 1974) it has been postulated that ascorbic acid is intimately involved in iron transport (Mazur, Green and Carleton, 1960; Osaki *et al.*, 1966). The increase in ascorbyl radical concentration in both spleen and liver might indicate reduction of Fe(III) to Fe(II) and possibly subsequent removal of iron from the tissues.

The e.s.r. results indicate that the observed changes in organs of leukaemic mice may be inter-related, representing different aspects of iron transport. Much work is still necessary to identify all the species involved and to determine their site of action within the cell. It is believed that e.s.r. can, in future, help to elucidate some of the biochemical events involved in the development of malignant disease. A change in one particular biochemical species may have many different causes but simultaneous examination of several species may give specific information. For example, changes in ceruloplasmin during the course of Hodgkin's disease have long found limited clinical application, but it now appears that simultaneous determination of ceruloplasmin and iron transferrin gives a more reliable guide to patient status (Foster, unpublished).

The author thanks Miss Justine Giron-Conland for her participation in the research and Mr R. Thompson for his invaluable technical assistance with the animals. The work was supported by the Medical Research Council and the Cancer Research Campaign.

### REFERENCES

AASA, R., MALMSTRÖM, B. G., SALTMAN, P. & VÄNNGÅRD, T. (1963) The Specific Binding of Iron (III) and Copper (II) to Transferrin and Conalbumin. *Biochim. biophys. Acta*, **75**, 203.

AISEN, P. (1974) The Role of Transferrin in Iron Transport. *Br. J. Haemat.*, **26**, 159.

AISEN, P., AASA, R., MALMSTRÖM, B. G. & VÄNNGÅRD, T. (1967) Bicarbonate and the Binding of Iron to Transferrin. *J. biol. Chem.*, **242**, 2484.

AISEN, P., PINKOWITZ, R. A. & LEIBMAN, A. (1973) EPR and Other Studies of the Anion-binding Sites of Transferrin. *Ann. N.Y. Acad. Sci.*, **222**, 337.

ANDRÉASSON, L-E. & VÄNNGÅRD, T. (1970) Evidence of a Specific Copper (II) in Human Ceruloplasmin as a Binding Site for Inhibitory Anions. *Biochim. biophys. Acta*, **200**, 247.

BEINERT, H. & PALMER, G. (1965) Contributions of EPR Spectroscopy to our Knowledge of Oxidative Enzymes. In *Advances in Enzymology*. Vol. 27. Ed. F. F. Nord. New York: J. Wiley and Sons. p. 105.

BLUMBERG, W. E. (1967) The EPR of high Spin $Fe^{3+}$ in Rhombic Fields. In *Magnetic Resonance in Biological Systems*. Eds A. Ehrenberg, B. G. Mälmström and T. Vänngård. Oxford: Pergamon Press. p. 119.

COMMONER, B., TOWNSEND, J. & PAKE, G. E. (1954) Free Radicals in Biological Materials. *Nature, Lond.*, **174**, 689.

COMMONER, B., WOOLUM, J. C., SENTURIA, B. H. & TERNBERG, J. L. (1970) The Effects of 2-acetyl-aminofluorene and Nitrite on Free Radicals and Carcinogenesis in Rat Liver. *Cancer Res.*, **30**, 2091.

DODD, N. J. F. (1975) ESR Study of Changes during the Development of a Yoshida Sarcoma. I. Paramagnetic Metal Ions. In preparation.

DODD, N. J. F. & GIRON-CONLAND, J. M. (1975) ESR Studies of Changes during the Development of a Mouse Myeloid Leukaemia. II. The Ascorbyl Radical. In preparation.

DRISCOLL, D. H., DETTMER, C. M., WALLACE, J. D. & NEAVES, A. (1967) Variation of ESR Signal Amplitude with Duration of Tumour Growth. *Curr. Mod. Biol.*, I, 275.

FOSTER, M. A. & HUTCHISON, J. M. S. (1974) The Origin of an ESR Signal at $g = 2 \cdot 03$ from Normal Rabbit Liver and the Effects of Nitrites upon it. *Phys. med. Biol.*, **19**, 289.

FOSTER, M. A., POCKLINGTON, T., MILLER, J. D. B. & MALLARD, J. R. (1973) A Study of Electron Spin Resonance Spectra of Whole Blood from Normal and Tumour Bearing Patients. *Br. J. Cancer*, **28**, 340.

FRIEDEN, E. (1971) Ceruloplasmin, a Link between Copper and Iron Metabolism. In *Bioinorganic Chemistry*. Ed. R. F. Gould. Advances in Chemistry Series, 100. Washington: American Chemical Society. p. 292.

FRIEDEN, E., McDERMOTT, J. A. & OSAKI, S. (1965) The Catalytic Activity of Ceruloplasmin and its Inhibition. In *Oxidases and Related Redox Systems*. Vol. I. Eds T. E. King, H. S. Mason and M. Morrison. New York: John Wiley and Son Inc. p. 240.

HOLLOCHER, T. C., SOLOMON, F. & RAGLAND, T. E. (1966) A Superfine Interaction involving $^{33}S$ in the Iron-containing Proteins of *Azobacter vinelandii*. *J. biol. Chem.*, **241**, 3452.

HRGOVCIC, M., TESSMER, C. F., MINCKLER, T. M., MOSIER, B. & TAYLOR, G. (1968) Serum Copper Levels in Lymphoma and Leukemia. *Cancer, N.Y.*, **21**, 743.

HRGOVCIC, M., TESSMER, C. F., THOMAS, F. B., FULLER, L. M., GAMBLE, J. F. & SHULLENBERGER, C. C. (1973*a*) Significance of Serum Copper Levels in Adult Patients with Hodgkin's Disease. *Cancer, N.Y.*, **31**, 1337.

HRGOVCIC, M., TESSMER, C. F., THOMAS, F. B., ONG, P. S., GAMBLE, J. F. & SHULLENBERGER, C. C. (1973b) Serum Copper Observations in Patients with Malignant Lymphoma. *Cancer, N.Y.*, **32**, 1512.

HRGOVCIC, M., TESSMER, C. F., BROWN, B. W., WILBUR, J. R., MUMFORD, D. M., THOMAS, F. B., SHULLENBERGER, C. C. & TAYLOR, G. (1973c) Serum Copper Studies in the Lymphomas and Acute Leukemias. In *Progress in Clinical Cancer*. Vol. V. Ed. I. M. Ariel. New York: Grune and Stratton Inc. p. 121.

HUGHES, N. R. (1972) Serum Transferrin and Ceruloplasmin Concentrations in Patients with Carcinoma, Melanoma, Sarcoma and Cancers of Haematopoietic Tissues. *Aust. J. exp. Biol. med. Sci.*, **50**, 97.

HUTCHISON, J. M. S., FOSTER, M. A. & MALLARD, J. R. (1971) Description of Anomalous ESR Signals from Normal Rabbit Liver. *Phys. med. Biol.*, **16**, 655.

JELLIFFE, A. M. (1973) Value of Fluctuations in the Serum Copper Level in the Control of Patients with Hodgkin's Disease. *Natn. Cancer Inst. Monog.*, **36**, 325.

KESSLER, D. L., JOHNSON, J. L., COHEN, H. J. & RAJAGOPALAN, K. V. (1974) Visualization of Hepatic Sulphite Oxidase in Crude Tissue Preparations by Electron Paramagnetic Resonance Spectroscopy. *Biochim. biophys. Acta*, **334**, 86.

MAILER, C., SWARTZ, H. M., KONIECZNY, M., AMBEGAONKAR, S. & MOORE, V. L. (1974) Identity of the Paramagnetic Element found in Increased Concentrations in Plasma of Cancer Patients and its Relationship to Other Pathological Processes. *Cancer Res.*, **34**, 637.

MALLARD, J. R. & KENT, M. (1966) Electron Spin Resonance in Surviving Rat Tissues. *Nature, Lond.*, **210**, 588.

MALLARD, J. R. & KENT, M. (1969) Electron Spin Resonance in Biological Tissues. *Phys. med. Biol.*, **14**, 373.

MAZUR, A., GREEN, S. & CARLETON, A. (1960) Mechanism of Plasma Iron Incorporation into Hepatic Ferritin. *J. biol. Chem.*, **235**, 595.

McDERMOTT, J. A., HUBER, C. T., OSAKI, S. & FRIEDEN, E. (1968) Role of Iron in the Oxidase Activity of Ceruloplasmin. *Biochim. biophys. Acta*, **151**, 541.

NEBERT, D. W. & MASON, H. S. (1963) An Electron Spin Resonance Study of Neoplasms. *Cancer Res.*, **23**, 833.

OSAKI, S., JOHNSON, D. A. & FRIEDEN, E. (1966) The Possible Significance of the Ferrous Oxidase Activity of Ceruloplasmin in Normal Human Serum. *J. biol. Chem.*, **241**, 2746.

OWEN, J. A. (1967) Effects of Injury on Plasma Proteins. In *Advances in Clinical Chemistry*. Vol. IX. New York: Academic Press. p. 1.

PEISACH, J. & BLUMBERG, W. E. (1970) Electron Paramagnetic Resonance Study of the High- and Low-spin Forms of Cytochrome $P_{450}$ in Liver and in Liver Microsomes from a Methylcholanthrene Treated Rabbit. *Proc. natn. Acad. Sci. U.S.A.*, **67**, 172.

PEISACH, J., BLUMBERG, W. E., OGAWA, S., RACHMILEWITZ, E. A. & OLTZIK, R. (1971a) The Effects of Protein Conformation on the Heme Symmetry in High-spin Ferric Heme Proteins as Studied by EPR. *J. biol. Chem.*, **246**, 3342.

PEISACH, J., OLTZIK, R. & BLUMBERG, W. E. (1971b) The Electron Paramagnetic Resonance of Molybdenum in Rat Liver and in Rat Liver Mitochrondria. *Biochim. biophys. Acta*, **253**, 58.

PRICE, E. M. & GIBSON, J. F. (1972) A Re-interpretation of Bicarbonate-free Ferric Transferrin EPR Spectra. *Biochem. biophys. Res. Commun.*, **46**, 646.

SAPRIN, A. N., KLOCHKO, E. V., KRUGLYAKOVA, K. E., CHIBRIKIN, V. M. & EMANUEL, N. M. (1966a) Kinetic Patterns of Change in the Content of Free Radicals on Malignant Growth and the Effects of Inhibitors of Radical Processes. *Biofizika*, **11**, 443.

SAPRIN, A. N., MINENKOVA, E. A., NAGLER, L. G., KOPERINA, E. V., KRUGLYAKO, S. A., KRUGLYAKOVA, K. E., VERMEL, E. M. & EMANUEL, N. M. (1966b) Kinetics of Change in the Content of Free Radicals with Development of Ascites Sarcoma 37. *Biofizika*, **11**, 616.

SAPRIN, A. N. NAGLER, L. G., KOPERINA, E. V., KRUGLYAKOVA, K. E. & EMANUEL, N. M. (1966c) Kinetics of Change in the Content of Free Radicals in the Blood and Organs of Mice with Experimental Leukaemia. II. *Biofizika*, **11**, 706.

SWARTZ, H. M., MAILER, C., AMBEGAONKAR, S., ANTHOLINE, W. E., McNELLIS, D. R. & SCHNELLER, S. J. (1973) Paramagnetic Changes during Development of a Transplanted AKR/J Leukemia in Mice as Measured by Electron Spin Resonance. *Cancer Res.*, **33**, 2588.

SWARTZ, H. M. & WIESNER, J. (1972) Radiation Effects on Plasma Electron-spin-resonance (ESR) Spectra of Cancer Patients. *Radiology*, **104**, 209.

TANAKA, T. (1969) Studies on the Development of Murine Leukaemia. *Ph.D. thesis, University of Manchester*.

TANAKA, T. & CRAIG, A. W. (1970) Differences in the Potential Transplantability of Murine lymphocytic and Myeloid Leukaemias. *Rev. Eur. Étud. clin. Biol.*, **15**, 505.

TESSMER, C. F., HRGOVCIC, M., BROWN, B. W., WILBUR, J. & THOMAS, F. R. (1972) Serum Copper Correlations with Bone Marrow. *Cancer, N.Y.*, **29**, 173.

TESSMER, C. F., HRGOVCIC, M., THOMAS, F. B., FULLER, L. M. & CASTRO, J. R. (1973a) Serum Copper as an Index of Tumor Response to Radiotherapy. *Radiology*, **106**, 635.

TESSMER, C. F., HRGOVCIC, M. & WILBUR, J. (1973b) Serum Copper in Hodgkin's Disease in Children. *Cancer, N.Y.*, **31**, 303.

TORMEY, D. C., IMRIE, R. C. & MUELLER, G. C. (1972) Identification of Transferrin as a Lymphocyte Growth Promoter in Human Serum. *Expl cell Res.*, **74**, 163.

TORMEY, D. C. & MUELLER, G. C. (1972) Biological Effects of Transferrin on Human Lymphocytes *in vitro*. *Expl cell Res.*, **74**, 220.

UPTON, A. C., JENKINS, V. K. & CONKLIN, J. W. (1964) Myeloid Leukemia in the Mouse. *Ann. N.Y. Acad. Sci.*, **114**, 189.

VÄNNGÅRD, T. (1974) Copper Proteins. In *Biological Applications of Electron Spin Resonance*. Eds H. M. Swartz, J. R. Bolton and D. C. Borg. New York: Wiley-Interscience. p. 411.

VITHAYATHIL, A. J., TERNBERG, J. L. & COMMONER, B. (1965) Changes in Electron Spin Resonance Signals of Rat Liver during Chemical Carcinogenesis. *Nature, Lond.*, **207**, 1246.

WARREN, R. L., JELLIFFE, A. M., WATSON, J. V. & HOBBS, C. B. (1969) Prolonged Observations on Variations in the Serum Copper in Hodgkin's Disease. *Clin. Radiol.*, **20**, 247.

WOOLUM, J. C., TIEZZI, E. & COMMONER, B. (1968) Electron Spin Resonance of Iron-nitric Oxide Complexes with Amino Acids, Peptides and Proteins. *Biochim. biophys. Acta*, **160**, 311.

ZSCHOCKE, R. H. & BEZKORAVAINY, A. (1974) Structure and Function of Transferrin II Transferrin and Iron Metabolism. *ArzneimFcrsch.*, **24**, 726.